# Assessment of food safety knowledge and practices among female canteen food handlers: a cross-sectional study

**DOI:** 10.3389/fpubh.2026.1925377

**Published:** 2026-09-28

**Authors:** Najla Albaridi, Jozaa Z ALTamimi, Nawal Albadr, Shaista Arzoo, Nora AlFaris

**Affiliations:** 1 Department of Health Science, College of Health and Rehabilitation, Princess Nourah bint Abdulrahman University, Riyadh, Saudi Arabia; 2 Department of Sports Health, College of Sports Sciences & Physical Activity, Princess Nourah bint Abdulrahman University, Riyadh, Saudi Arabia; 3 Department of Food Sciences and Nutrition, College of Food Science and Agriculture, King Saud University, Riyadh, Saudi Arabia

**Keywords:** canteen, cleaning and disinfection, food safety, health and personal hygiene, knowledge, university

## Abstract

**Background:**

Improper handling of food during preparation and serving is one of the major causes of food hygiene issues.

**Objectives:**

This cross-sectional study was conducted using a total population sampling method and aimed to evaluate the knowledge and practices of food service workers regarding the principles of food safety at the food court in Women’s only University.

**Materials and methods:**

Data were collected using a questionnaire by interviewing 118 workers at the food court and were analyzed using descriptive statistics and correlation analysis. The questionnaire included demographic information, education and training related questions and food safety awareness questions.

**Results:**

Food service workers scored 42.15 on the food safety awareness questionnaire, while the mean score was 9.69 (80.8%) for the knowledge-based questions, 9.37 (85.2%) for health and personal hygiene, 2.91 (72.8%) for cleaning and disinfection category and 20.18 (74.7%) for attitudes and temperature control related questions and statistically significant and positive correlations were noted among all four assessed domains.

**Conclusion:**

University should implement structured and frequent training programs for all food handlers focusing on cleaning, sanitization, and temperature. Additionally, routine supervision should also be enforced. Longitudinal and interventional studies including both males and females across different regions of Saudi Arabia are required to evaluate the long-term efficacy of food safety policies and training programs.

## 1 Introduction

Food safety is a global concern that affects public health across all ages, races, genders, and socioeconomic backgrounds (1). Safe food is “a food that appears consumable and has been handled in the correct way, in terms of handling, distribution and storage at appropriate temperature” (2). World Health Organization statistics indicate that one in nine people worldwide get sick from contaminated food each year, which leads to approximately 1.52 million deaths (3). There are several ways in which contaminants enter the food system, including cross-contamination, contaminated raw materials, improper food handling, insufficient personal hygiene, improper storage conditions and inadequate cooking (4). Even with the application of food quality management systems, outbreaks of foodborne diseases continue and are on the rise (5). To ensure food security in the population and promote public health, safe and nutritious food must be available (6).

Food handlers are a crucial part of commercial food management systems and can cause numerous foodborne illnesses if proper food safety practices are not followed (7). WHO defined food handlers as “those persons who in the course of their normal routine work, handle food or food items or those who may come into contact with handling of eating and drinking utensils not meant for their personal use” (8). A study by Campos et al. demonstrated that food handlers are a major cause of food contamination (9). In a review study, Kamboj et al. have shown that almost 39% foodborne illnesses are result of cross-contamination, around 45% occur due to inappropriate storage practices, while improper food storage is responsible for around 50% of foodborne illnesses (10).

In Saudi Arabia, people regularly consume food at restaurants and food courts, which increases the incidence of food poisoning. Many food-borne illness outbreaks have been reported in Saudi Arabia over the past decade. A study by Al-Goblan and Jahan analyzed 251 cases of foodborne illness reported during the summer of 2006 (11). Moreover, *Bacillus cereus* was responsible for an outbreak during the pilgrimage (Hajj) in 2006 in a group of Saudi soldiers, who consumed contaminated rice (12). Another study conducted by Al-Honazil et al. reported an outbreak in Bisha city that included 55 cases of food poisoning (13). In a recent study in April 2024, Riyadh, Saudi Arabia was the site of a substantial foodborne botulism outbreak, where the Ministry of Health reported 75 cases, with 69 cases among citizens and 6 cases among residents and of these, 50 were laboratory-confirmed. The outbreak resulted in 43 recoveries, 11 hospitalizations, 20 admissions to intensive care units and one fatality (14, 15).

Food safety and hygiene are important aspects in the food industry and concerns related to food hygiene and its safety in the Kingdom of Saudi Arabia are covered by the organizations namely “Saudi Food and Drug Authority (SFDA) and Ministry of Municipality and Rural Affairs”. Former (SFDA) is a central authority that was established to ensure the food safety of both locally and imported foods and to make sure that they adhere to recognized national and international standards while later has legitimate and execution roles in the food safety sector (16). Food safety refers to the fundamental processes and rules that must be adhered to in order to ensure that food is safe and suitable for human consumption during production, processing, storage, and distribution. Knowledge, attitude, and practice are the three main elements influencing food poisoning outbreaks involving food handlers (17).

Many studies have been conducted to examine the knowledge, attitudes, and behaviors (KAP) related to food safety among food handlers in different nations to assess how well these standards are being followed. In a study carried out in kitchen facilities in North Jakarta, Indonesia, Putri and Susanna reported a significant positive correlation between food handlers’ knowledge and attitudes regarding food safety, but no significant correlation was found between knowledge and attitudes and food handling practices (18). In another study carried out in school kitchens in Espírito Santo, Brazil, da Vitória et al. observed that while food safety knowledge scores were associated with work experience and training time, attitudes were linked to education and training time, and practices were primarily influenced by attitudes (19). Abdel-hafez et al. in their study have shown lower level of food safety knowledge regarding food-borne pathogens among workers without educational courses and training, while Al-Mohaithef et al. explained the need for training that could have a positive impact in food handler knowledge, attitude, and practices (20, 21). Despite existing research on food safety, hardly few studies have evaluated the food safety knowledge among female canteen food handlers in Riyadh, highlighting a clear gap that the present study aims to address. So, this study aimed to evaluate the knowledge and practices of food service workers regarding the principles of food safety at the food court in Women’s University in Riyadh.

## 2 Materials and methods

### 2.1 The study design and target participants

A cross-sectional study was conducted using total population sampling method that assessed the food safety knowledge and practices of all eligible food service workers in women’s University in Riyadh, Saudi Arabia. All Canteen employees who directly handle the food were invited to participate.

#### 2.1.1 Inclusion criteria

All female food service workers aged 18 years or older who were directly involved in food handling, whether in preparation, cooking, heating, or serving, were available during the study (data collection) period and willing to participate in the study (proficient in either Arabic or English) were included in the study.

#### 2.1.2 Exclusion criteria

Female workers who were not directly involved in food handling, such as cashiers or cleaning staff or unavailable during the data collection period, not ready to provide consent. Moreover, those who fulfilled the inclusion criteria but did not complete the questionnaire were also excluded from the study.

#### 2.1.3 Sample size

All food service handlers, a total of 154 participants, were initially enrolled in the study. However, 36 questionnaire were excluded due to incomplete responses, resulting in a final valid sample of 118 participants (response rate: 76.6%).

### 2.2 Questionnaire design

A questionnaire was designed to collect data which was based on previous food safety studies (18, 22, 23), and translation to Arabic and translation back to English were made to ensure the suitability for all target population. The validity and reliability of the questionnaire were confirmed by presenting it to food safety specialists and then necessary revisions were made. The questionnaire was tested on a pilot sample of about 30 respondents before data collection began. Based on their responses, some of the questionnaire translated items that were unclear were modified, and Cronbach’s alpha coefficient was calculated for the questionnaire items. The Cronbach’s alpha coefficient was 0.9 for knowledge-based questions, 0.85 for both health and personal hygiene questions and for cleaning and disinfection questions and 0.9 for attitudes and temperature control of food questions.

The first section included demographic information from each participant, including age, nationality, educational level, years of experience in the food service industry, and occupational classification. The second section focused on participants’ education and training, particularly on food safety. This included food safety instruction received at the start of employment at the student services center, participation in food safety awareness lectures during employment, and attendance at annual training courses or workshops related to food safety. The third section of the questionnaire assessed food safety awareness through 54 questions organized into four domains: 12 questions on knowledge of food safety, 11 on health and personal hygiene, 4 on cleaning and disinfection, and 27 on attitudes and temperature control. Each correct response was scored 1 point, and incorrect responses were scored 0 points, resulting in a maximum possible score of 54 points. Three levels of food safety awareness were established using a 54-point scoring system: excellent awareness (score >38; >70%), moderate awareness (score 27–38; 50–70%), and poor awareness (score <27; <50%), consistent with the classification criteria established by Ovai et al. (24).

### 2.3 Institutional review board statement

The study was approved by the Scientific Research Ethics Committee at Princess Nourah bint Abdulrahman University (IRB Log Number: 18-0012). The objective of the study was explained to all participants, and written informed consent was obtained from each respondent. The study adhered to the principle of voluntary participation. The participant’s confidentiality was maintained by removing personal identifiers and anonymizing data and no information that can disclose the identity of participant was disclosed in any report.

### 2.4 Statistical analysis

All statistical analyses were carried out with SPSS (version 15, IBM Corp., Armonk, NY, USA). Descriptive statistics (frequency, percentages, mean and standard deviation (SD)) were applied. Spearman’s correlation was used to study the relationship between food safety awareness and demographic characteristics and training and also applied to assess the association between different food safety awareness items. For Spearman’s correlation, the variables were ordinal and a monotonic relationship between variables was assessed. A significant result means that the *p*-value for the hypothesis tests is less than 0.05.

## 3 Results

### 3.1 Demographic characteristics of the study participants

Table 1 depicts the demographic characteristics of the study participants. The mean age of the participants was 32.73 ± 7.92 years. In terms of nationality, the majority were non-Saudi (59.3%). Regarding educational level, 51.7% of participants reported having “other” (primary, secondary or intermediate level education) types of education, 28.8% held a diploma, and 19.5% had a bachelor’s degree. None of the participants held a master’s or doctorate degree. Most (94.9%) of them had less than 10 years of experience, while 3.4% had experience of 10–20 years. The majority of participants were classified as food workers (51.7%), followed by those in the “other” (site supervisor, supervisor, or food handels) category (39.8%) and a smaller portion were cooks (8.5%).

**Table 1:** Sociodemographic characteristics of the study participants (*N* = 118).

Demographic data	N (%)
Age*	32.73 ± 7.92
Nationality	
Saudi	48(40.7)
Non-Saudi	70(59.3)
Educational level	
Diploma	34(28.8)
Bachelor’s degree	23(19.5)
Master or Doctorate	0(0)
Other	61(51.7)
Years of work experience	
≤10 years	112(94.9)
10 to 20 years	4(3.4)
≥20 years	2(1.7)
Professional classification	
Nutritionist	0(0)
Technician	0(0)
Cook	10(8.5)
Food worker	61(51.7)
Other	47(39.8)

Data are presented as frequency and percentage except for age* where data is presented as mean ± standard deviation.

### 3.2 Education and training of the study participants

The education and training of the study participants is shown in Table 2. Most participants (92.4%) reported receiving workplace food safety training. However, only 57.6% received training when starting work, and 53.4% reported receiving annual training.

**Table 2:** Education and training of the study participants (*N* = 118).

Training questions	Response	N (%)
Have you been trained in the food safety system at your work?	Yes	109 (92.4)
	No	9(7.6)
	Total	118(100.0)
Did you take food safety course when you start work?	Yes	68(57.6)
	No	50(42.4)
	Total	118(100.0)
Do you take food safety training courses at least once a year while you work?	Yes	63(53.4)
	No	55(46.6)
	Total	118(100.0)

Data are presented as frequency and percentage.

### 3.3 Participant responses to food safety awareness questions

Participant responses to food safety awareness questions are presented in Table 3. On average, food service workers scored 42.15 ± 14.60 out of 54 questions. The mean score for the 12 knowledge-based questions was 9.69 ± 2.73, while the mean score for the 11 health and personal hygiene questions was 9.37 ± 2.43. For the cleaning and disinfection category (4 questions), the mean score was 2.91 ± 1.23. Additionally, participants had a mean score of 20.18 ± 8.21 on the 27 questions related to attitudes and temperature control of food. Overall, food safety awareness was relatively high, particularly in the domains of knowledge and personal hygiene. However, relatively lower levels of correct responses were observed for cleaning and disinfection and several temperature-control practices (Figure 1). The proportions of correct responses and their corresponding 95% confidence intervals are presented in Table 3. The lowest levels of correct responses were observed for questions related to biological contamination, food allergies, cross-contamination, proper cleaning and washing procedures, and optimal food storage.

**Table 3:** Participants’ responses to food safety awareness questions, including 95% confidence intervals for the proportion of correct responses (*N* = 118).

S. No.	Questions	Correct response N (%)	Incorrect response N (%)	95% CI (%)
Knowledge based questions				
1	HACCP is a system for analyzing various sources of risk and analyzing critical control points to ensure food safety by identifying sources of risk	86 (72.9)	32(27.1)	64.2–80.1
2	A written procedure for food storage should be always available for staff	107(90.7)	11(9.3)	84.1–94.7
3	Hazards can be safe if you follow the basic food safety guidelines	103(87.3)	15(12.7)	80.1–92.1
4	There is a regular internal food safety inspection	106(89.9)	12(10.2)	83.1–94.1
5	All Staff should get training in food safety	109(92.4)	9(7.6)	86.1–95.9
6	The presence of detergents in the food preparation area is a type of chemical contamination	106(89.8)	12(10.2)	83.1–94.1
7	Broken glass, jewelry, Band-Aids, staples, stick pins, or fingernails all are physical contaminants	105(89.0)	13(11.0)	82.1–93.4
8	Biological hazards are the most cause of food problems*	82(69.5)	36(30.5)	60.7–77.1
9	Food allergy team should include supervisors, diet technicians, cooks, food service workers and tray checkers*	73(61.9)	45(38.1)	52.9–70.1
10	All meals for patients with food allergies should be prepared in separate cooking tools/ kitchen to assure their safety	95(80.5)	23(19.5)	72.4–86.6
11	Carrying a special order on a tray with other food items can cause cross contact*	74(62.7)	44(37.3)	53.7–70.9
12	The name of the person who is responsible for answering the food allergic patient’s questions about food choices and menu selection should be mentioned in the written procedure for handling food allergies	97(82.2)	21(17.8)	74.3–88.1
	Total %	80.73	19.27	
	Mean ± S. D.	**9.69 ± 2.73**		
Questions related to health and personal hygiene				
13	Hand sanitizers cannot be used in place of hand washing to clean hands before handling food	92(78.0)	26(22.0)	69.7–84.5
14	Wash your hands before and after touching unwrapped raw food	110(93.2)	8(6.8)	87.2–96.5
15	Wash your hands before and after touching unwrapped cooked food	111(94.1)	7(5.9)	88.3–97.1
16	Wash your hands before and after Handling raw foods (including meats, eggs, and fresh fruits and vegetables	110(93.2)	8(6.8)	87.2–96.5
17	The preparation sink should not be used for hand washing	97(82.2)	21(17.8)	74.3–88.1
18	If you work with food, your own personal habits can affect the food safety	85(72.0)	33(28.0)	63.3–79.3
19	Jewellery should not be worn while preparing food	94(79.7)	24(20.3)	71.5–85.9
20	Using cap and protective gloves reduces the risk of food contamination	102(86.4)	16(13.6)	79.1–91.5
21	Wear disposable gloves when you have a cut or sore	99(83.9)	19(16.1)	76.2–89.4
22	Food service staff with abrasion or cuts on hands should not touch unwrapped food	102(86.4)	16(13.6)	79.1–91.5
23	If you feel symptoms of vomiting or diarrhea at work, you should stop work immediately	104(88.1)	14(11.9)	81.1–92.8
	Total %	85.2	14.8	
	Mean ± S. D.	**9.37 ± 2.43**		
Questions related to cleaning and disinfection-related				
24	Cleaning agents should be stored away from or below any food or food-contact surfaces	98(83.1)	20(16.9)	75.3–88.8
25	A towel should be used to dry dishes or to set dishes upon to dry	85(72.0)	33(28.0)	63.3–79.3
26	Always Spray or scrape off all food leftovers*	80(67.8)	38(32.2)	58.9–75.6
27	The sanitizer should be warm water with some soap added*	80(67.8)	38(32.2)	58.9–75.6
	Total %	72.67	27.33	
	Mean ± S. D.	**2.91 ± 1.23**		
Questions related to attitudes and temperature control of food				
28	Handling unwrapped food with cuts or abrasions on hands can cause contamination	98(83.1)	20(16.9)	75.3–88.8
29	Sharing of utensils for raw and uncooked foods can cause cross-contamination	93(78.8)	25(21.2)	70.6–85.2
30	Wipe cloths should be used around raw meat separate from other foods*	75(63.6)	43(36.4)	54.6–71.7
31	Potentially hazardous food must be maintained between 4 and 60 °C*	82(69.5)	36(30.5)	60.7–77.1
32	Humidity, temperature, and food pH are important factors affecting the reproduction of microorganisms in food	98(83.1)	20(16.9)	75.3–88.8
33	Food thermometer should be used to make sure that the food is cooked to a safe and to measure minimum internal temperature	106(89.8)	12(10.2)	83.1–94.1
34	Food can be kept at the temperature danger zone for a maximum of two hours while being prepared*	77(65.3)	41(34.7)	56.3–73.2
35	Food that left in the danger zone for 2 h cannot be reheated or cooled for hot or cold holding and must be eaten within the next 2 h or thrown away	93(78.8)	25(21.2)	70.6–85.2
36	Frozen foods must be left to thaw at room temperature (on the counter)*	76(64.4)	42(35.6)	55.4–72.5
37	Keep the meat cooked at a temperature higher than 64 °C	91(77.1)	27(22.9)	68.8–83.8
38	Raw meat should be stored on bottom shelf of refrigerator to avoid dripping*	82(69.5)	36(30.5)	60.7–77.1
39	Raw food should be kept separate from cooked food	88(74.6)	30(25.4)	66.0–81.6
40	Unused defrosted food should be refrozen immediately	85(72.0)	33(28.0)	63.3–79.3
41	Food must be cooled quickly to avoid being in the temperature danger zone for too long*	81(68.6)	37(31.4)	59.8–76.3
42	When it is necessary to cool foods for reheating later you must follow the cooling curve to avoid germ growth in the food	93(78.8)	25(21.2)	70.6–85.2
43	Thermometers should be used to check the internal temperatures of foods when being cooked, cooled, and reheated	90(76.3)	28(23.7)	67.8–83.0
44	It is important to check the temperature of the refrigerator/freezer regularly to reduce the risk of food spoilage	101(85.6)	17(14.4)	78.1–90.8
45	Improper storage of foods may be cause of health hazards to consumers	98(83.1)	20(16.9)	75.3–88.8
46	Use separate kitchen utensils to prepare raw and cooked food	93(78.8)	25(21.2)	70.6–85.2
47	Raw and ready-to-eat foods separate	97(82.2)	21(17.8)	74.3–88.1
48	Harmful bacteria are destroyed when food is cooked to proper temperatures	92(78.0)	26(22.0)	69.7–84.5
49	Raw eggs contain a microorganism that could cause food poisoning*	77(65.3)	41(34.7)	56.3–73.2
50	Raw poultry can pose a significant risk of salmonella poisoning*	82(69.5)	36(30.5)	60.7–77.1
51	Ready-to-eat foods are foods which will not be cooked after handling them and before being eaten	85(72.0)	33(28.0)	63.3–79.3
52	Potentially hazardous foods that have been cooked or prepared (e.g., meats, salads, cooked vegetables) must be eaten or discarded within 7 days*	63(53.4)	55(46.4)	44.4–62.1
53	Potentially hazardous foods prepared on-site, held for more than 24 h, and refrigerated must be labelled with the date prepared or the date to throw away	98(83.1)	20(16.9)	75.3–88.8
54	Meat, poultry, fish, eggs and their products should be stored cold at temperature lower than 4 °C	87(73.7)	31(26.3)	65.1–80.8
	Total %	74.74	25.26	
	Mean ± S. D.	20.18 ± 8.21		
	**Mean ± S. D. (Total questions)**	**42.15 ± 14.60**		

Data are presented as frequency and percentage. The 95% confidence intervals (CI) were calculated for the proportion of correct responses using the Wilson method. Bold values indicate the mean ± standard deviation and the corresponding 95% confidence interval.

**Figure 1:**
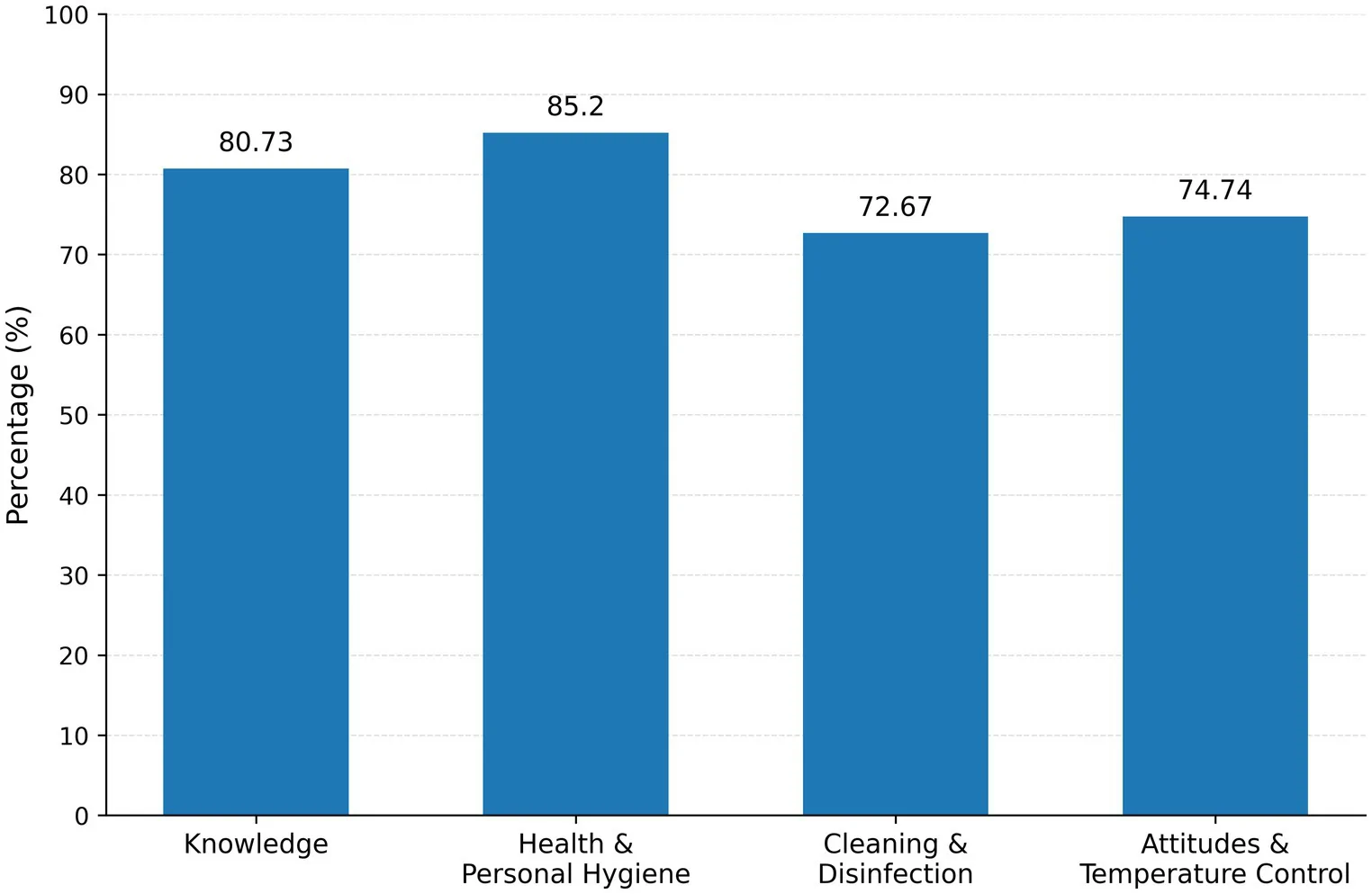
Participant’s awareness on knowledge, health and personal hygiene, cleaning and disinfection and attitude and temperature control-based questions.

### 3.4 Level of food safety awareness among study participants

Table 4 presents the level of food safety awareness among the study participants. Most participants demonstrated excellent awareness, with 78.81% (*n* = 93; 95% CI: 70.6–85.2%) correctly answering more than 70% of the 54 questions. A good level of awareness was observed among 5.08% of participants (*n* = 6; 95% CI: 2.4–10.6%), who scored between 50 and 70%. In contrast, 16.10% of participants (*n* = 19; 95% CI: 10.6–23.6%) demonstrated weak awareness, with scores below 50%.

**Table 4:** Level of food safety awareness among study participants (*N* = 118).

Degree of awareness	N (%)	95% CI (%)
More than 70%; Excellent awareness (>38 correct answers)	93(78.81)	70.6–85.2
50–70%; Good awareness (27–38 correct answers)	6(5.08)	2.4–10.6
Less than 50%; Weak awareness (<27 correct answers)	19(16.10)	10.6–23.6

Data are presented as frequency and percentage. The 95% confidence intervals (CI) for the proportions were calculated using the Wilson score method.

### 3.5 Correlation between food safety awareness levels and participants’ demographic characteristics and training

Table 5 depicts the results of Spearman correlation analysis between the level of food safety awareness and the demographic data and training of the participants. A statistically significant but weak negative correlation was found between educational level and food safety knowledge (*r* = −0.199, *p* = 0.031) and between professional classification and attitude related questions. Spearman’s correlation analysis showed positive association between food safety training and various food safety awareness domains. We observed that food safety training at work was significantly associated with knowledge based questions (*r* = 0.210, *p* = 0.022), and health and personal hygiene questions (*r* = 0.253, *p* = 0.006). Similarly, food safety courses provided at the start of work were associated with knowledge (*r* = 0.331, *p* < 0.001), health and personal hygiene (*r* = 0.342, *p* < 0.001) and attitudes and temperature control of food questions (*r* = 0.297, *p* = 0.001). In addition, attending food safety training courses at least once a year was positively associated with knowledge (*r* = 0.194, *p* = 0.036).

**Table 5:** Correlation between food safety awareness levels and participants demographic characteristics and training.

Demographic	Knowledge questions		Health and personal hygiene questions		Cleaning and disinfection questions		Attitudes and Temperature Control of Food questions	
	Sp. Cor.	Sig.	Sp. Cor.	Sig.	Sp. Cor.	Sig.	Sp. Cor.	Sig.
Age	0.122	0.189	0.091	0.327	0.048	0.609	0.144	0.121
Nationality	0.163	0.078	−0.020	0.834	−0.144	0.121	0.036	0.695
Educational level	−0.199	0.031*	−0.164	0.076	−0.053	0.566	−0.131	0.156
Years of work experience	0.104	0.264	−0.009	0.922	0.022	0.815	−0.074	0.428
Professional Classification	−0.044	0.638	0.016	0.860	−0.041	0.662	−0.214	0.020*
Training questions								
Have you been trained on the food safety system at your work?	0.210	0.022*	0.253	0.006*	0.076	0.415	0.122	0.188
Did you take food safety course when you start work?	0.331	0.0001**	0.342	0.0001**	0.086	0.357	0.297	0.0001**
Do you take food safety training courses at least once a year while you work?	0.194	0.036*	0.122	0.188	0.167	0.070	0.074	0.424

Model: Spearman correlation; Bold values indicate statistically significant *p*-values; *p < 0.05, *p* < 0.001**; Sp. Cor.: Spearman’s correlation.

### 3.6 Correlation between various levels of food safety awareness standards question

Table 6 depicts statistically significant and positive correlations among all four assessed domains (knowledge, health and personal hygiene, cleaning and disinfection, attitudes and temperature control of food) of food safety.

**Table 6:** Correlation between the levels of awareness of the participants according to the food safety standards questions.

Food safety standards questions	Knowledge questions		Health and personal hygiene questions		Cleaning and disinfection questions		Attitudes and temperature control of food questions	
	Sp. Cor.	Sig.	Sp. Cor.	Sig.	Sp. Cor.	Sig.	Sp. Cor.	Sig.
Knowledge questions			0.663	0.0001**	0.493	0.0001**	0.575	0.0001**
Health and personal hygiene questions	0.663	0.0001**			0.381	0.0001**	0.485	0.0001**
Cleaning and disinfection questions	0.493	0.0001**	0.381	0.0001**			0.576	0.0001**
Attitudes and temperature control of food questions	0.575	0.0001**	0.485	0.0001**	0.576	0.0001**		

Bold values indicate statistically significant *p*-values; *p < 0.05, *p* < 0.001**.

## 4 Discussion

The present study analyzed the food safety knowledge of female food service workers. Most of the participants in this study had fewer than 10 years of experience aligning with findings from earlier studies done by Abdelhafez, 2013 and Teffo and Tabit, 2020 (20, 25). A good level of knowledge regarding health and personal hygiene and knowledge-based questions was demonstrated by the workers in the present study. But, the knowledge related to temperature control of food and cleaning and disinfection was relatively low. The findings of the current study are consistent with previous studies reported in Saudi Arabia and other countries. For example, a study conducted among food handlers in restaurants of King Saud University, Riyadh, Saudi Arabia, demonstrated that, the majority of food handlers had good food and hygiene practices and good understanding of food safety related to hygiene, foodborne illness, and cross-contamination, but their knowledge about the temperature and time control was least (23). Similarly, a study based on hospital food service staff in Al Madinah, Saudi Arabia, reported generally good levels of food safety knowledge and practices. Knowledge of food handlers was greatest for cross-contamination but food storage was less well understood highlighting the significance of education and training of food service staff in improving knowledge (26). A recent review study by Aljasir demonstrated that food handlers across the Gulf, including Saudi Arabia, have gaps in both knowledge and practices regarding food safety. The review also highlighted the importance of education and training in improving food safety knowledge and practices (27).

In line with our study, Nee and Sani in their study on food handlers from two residential colleges’ cafeterias and one Faculty of Science and Technology’s canteen reported good knowledge of personal hygiene (93.9%) and foodborne illness definitions (73.9%), whereas, only 28% of respondents had sufficient knowledge of food storage and preparation temperatures. Similarly, positive attitudes toward food safety and hygiene (76.9%) and foodborne disease prevention and control (70.8%) were reported (28). The similarity in the low level of knowledge of temperature control suggests that this may be an area for greater emphasis in food safety training. In another study by Sani and Siow which was conducted at the main campus of University Kebangsaan Malaysia (UKM), the respondents demonstrated good knowledge scores (19.68 ± 3.87) regarding personal hygiene, foodborne diseases, and temperature control; positive attitudes (89.26 ± 8.66) toward safe food handling; and good practices (90.02 ± 8.23), particularly in proper hand washing, use of gloves during food preparation and prevention of food hazards (29). However, another study by Webb and Morancie reported that 63.5% of university food service workers had limited overall knowledge, while 79% demonstrated adequate understanding of hygiene practices, yet only 33.9% possessed sufficient awareness of proper food temperature, time and control methods (30). In the present study, also lower cleaning and temperature control knowledge was observed which may be due to lack of detailed and frequent training on cleaning procedures, as well as a lack of comprehension of technical food safety concepts like refrigeration temperature, cooking and storage temperature, and thawing process. In addition to this, prioritizing quick service during peak hours and a lack of supervision contribute as well to lower cleaning and temperature control knowledge. A study on food service workers at a university in Kuala Lumpur found that although 61% had satisfactory food safety knowledge, this did not translate into proper practices, as hand examinations showed 65% had bacterial infections and 48% tested positive for Salmonella (31). According to Sihombing et al. workers from small food companies studied in Maluku-Indonesia demonstrated an acceptable level of food security and hygiene knowledge (32). The consistency of the above findings implies that even when food handlers may have sufficient awareness of general hygiene practices, certain aspects of temperature management may still be poorly comprehended. This emphasizes the significance of temperature management in food safety instruction.

Moreover, Kubde et al. noted that most food workers had general food safety knowledge and very good practices, although most of these workers were not trained on food safety principles and the bulk of their food safety information originated from the media (33). Asekun-Olarinmoye et al. reported that workers at the university cafeteria in Babcock have a satisfactory level of knowledge and practices towards food safety, but these subjects did not show good practices in environmental hygiene (34). This disparity shows that workers’ environmental hygiene practices need to be strengthened through focused interventions. It might be able to bridge the knowledge-to-practice gap and create a safer food handling environment by strengthening training programs and enforcing more stringent hygiene regulations. Research shows that food safety policies are linked to better practices, such as cleaning and sanitizing equipment properly, staying at home when ill, and properly datemarking on ready-to-eat foods. The development and execution of clear policies that specify when and how to perform hand hygiene can improve compliance (35).

We found positive associations between food safety training and the different food safety domains, which may be linked to a broader comprehension of food safety by the food service personnel. The association of knowledge, health and personal hygiene, cleaning and disinfections and attitudes and temperature control observed in our study indicates that training may be more effective in raising awareness of different areas of food safety than in providing knowledge alone. The result of this study is consistent with the findings of Al-Shabib et al. who reported a positive relationship between the knowledge and practice of food service employees, as well as their training, personal knowledge and cleanliness, and personal hygiene practices (22). Sani and Siow also found a positive correlation between the groups of questions related to the knowledge and practices on the prevention of food hazards (29). In contrast, Webb and Morancie reported that there was no relationship between the level of education and knowledge of food safety rules, while there was a relationship between experience and food safety, but training did not affect knowledge (30). A statistically significant but weak negative correlation was found between educational levels and knowledge. The high percentage of individuals categorized as having “Other” education, may have contributed to this outcome; as the specific type of education in this category were not mentioned, so this finding should be interpreted with caution. In the present study spearman’s correlation analysis revealed statistically significant positive relationships between food safety training (both at work and at the start of employment) and employees’ knowledge, health and personal hygiene. Attitudes and temperature control scores showed association with course at the start of work. It is important to note that, even though the correlations were not large, the consistent positive direction indicates that the presence of food safety training was associated with multiple aspects of food safety awareness among the workers. In the study of Adesokan et al. where the correlation between training, area and duration of training, and refresher training on food safety knowledge and practices was assessed, the authors found a correlation between training and knowledge and practices of food safety (36). The training also impacted on the knowledge of food safety and practices, while refresher training had a clear impact on the level of knowledge and practices of food service workers (36). Various studies conducted have also reported a correlation between education level and training experience on food safety knowledge and practices (32, 33).

The study has several limitations including a small sample size drawn from a single university, which may introduce a selection bias and restrict the generalizability of the findings. Another limitation is the lack of direct observational assessment of participant actual practices because the findings depend on self-reported data which again increases the risk of bias and the participants were gender restricted, i.e., only female participants were involved in the study. The “other” education category was not further specified, which limited the interpretation of the observed association with food safety awareness.

## 5 Conclusion and recommendations

This study assessed the food safety knowledge of workers in university food court. The results demonstrate high food safety awareness among food handlers, specifically in knowledge and personal hygiene, while comparatively lower scores in cleaning, disinfection, and temperature control indicate weaker practices in these areas. Therefore, universities should implement structured and strong training programs frequently for all food handlers focusing on cleaning, sanitization, and temperature. All food handlers should be provided with regular food safety training, at least once a year to refresh their knowledge and awareness, including practical demonstrations and periodic supervisory checks to reinforce proper practices and to identify areas where refresher training may be needed. Further research should be conducted across different universities in different regions of the country to capture more comprehensive, country-level understanding of food safety practices among food service workers on campus. To evaluate the long-term efficacy of food safety policies and training programs, longitudinal and interventional studies are also required. These initiatives would aid in the development of more focused, evidence-based strategies to improve food safety regulations across the country.

## Data Availability

The original contributions presented in the study are included in the article/supplementary material, further inquiries can be directed to the corresponding author.
